# Quality of Life and Appraisal Factors of Patients with Advanced Cancer and Their Family Caregivers

**DOI:** 10.21203/rs.3.rs-4915960/v1

**Published:** 2024-10-14

**Authors:** Jia Liu, Yuexia Zhang, Ting Guan, Xiaomeng Wang, Chunxuan Ma, Laurel Northouse, Lixin Song

**Affiliations:** The University of Texas Health Science Center at San Antonio; The University of Texas at San Antonio; Syracuse University; The University of Texas Health Science Center at San Antonio; The University of Texas Health Science Center at San Antonio; University of Michigan–Ann Arbor; The University of Texas Health Science Center at San Antonio

**Keywords:** Advanced cancer, Caregiver, Quality of life, Appraisal, Multi-level model

## Abstract

**Purpose:**

Few existing interventions have effectively improved the quality of life (QOL) for patients with advanced cancer and their caregivers, partly due to limited research on the factors associated with QOL. Guided by an adapted stress-coping model, this study aimed to examine the associations between the QOL of cancer patients and their caregivers and their primary and secondary appraisals. Primary appraisals involve perceptions and evaluations of advanced cancer and related caregiving, while secondary appraisals relate to their available resources and coping capabilities.

**Methods:**

Using multi-level modeling, we conducted a secondary analysis of the baseline data collected from a randomized clinical trial that examined the effects of a family-based, psychoeducational support program for patients with advanced cancer and their caregivers (N = 362 dyads).

**Results:**

The appraisal variables hypothesized in the adapted stress-coping model explained 74.14% of the variance in the QOL of patients with advanced cancer and their caregivers when controlling for demographics and other disease-related variables. Better QOL in patients and caregivers was associated with less negative appraisals of illness/caregiving, less uncertainty and hopelessness, less avoidant coping strategies, more family support, more health behaviors, higher self-efficacy, and more active coping strategies.

**Conclusion:**

Our study highlights the significant impact that advanced cancer has on patients and their caregivers’ perceptions, responses to the illness, and QOL. It also highlights that effective interventions may need to target illness/caregiving appraisals, uncertainty, hopelessness, family support, health behaviors, self-efficacy, and coping strategies tailored to patient and caregiver needs.

## Background

Quality of life (QOL) in patients with advanced cancer is an essential outcome in cancer care [1]. These patients experience various physical, functional, psychological, emotional, and social challenges triggered by symptoms such as pain, chronic fatigue, cachexia, and breathlessness, as well as distress and dire prognoses; this combination of factors significantly compromises their QOL [2]. Similarly, caregivers who provide intensive care and support to patients with deteriorating health face significant physical, emotional, social, and financial burdens. The thought of losing a loved one due to a poor prognosis further reduces caregivers’ QOL. Family caregivers often experience QOL levels similar to, or even worse than, those reported by cancer patients [1, 3], impairing their caregiving capacity and ultimately further decreasing patients’ QOL [4].

To manage symptom burden and distress and to improve their QOL, patients with advanced cancer and caregivers engage in complex and evolving appraisal processes. These processes involve perceiving and interpreting stressful situations and assessing their ability to cope with those stressors [5]. However, despite these efforts, managing the multifaceted challenges of advanced cancer remains difficult for patients and caregivers. As a result, the effectiveness of interventions to improve their QOL is crucial.

Most interventions aimed at improving the QOL for patients and caregivers have focused primarily on strategies for delivering supportive care related to activities of daily living [2, 6, 7], symptom management [2, 6–8], changing behaviors and thoughts [2, 7], managing burden and improving coping [2, 6–8], enhancing their sense of meaning [2], and promoting cancer communication with family and physicians about care planning [2, 6–8]. However, systematic reviews have shown that only a minor proportion of these existing interventions have effectively improved the QOL for patients and caregivers [2, 6, 8]. Furthermore, these studies used mixed study designs and modalities [2, 6].

Other interventions have been less effective due to a lack of theoretical underpinnings and a limited understanding of the care needed to improve QOL among patients and caregivers managing advanced cancer [2, 6, 8]. To date, limited research has thoroughly investigated the factors associated with QOL for patients with advanced cancer and their caregivers, which is critical for developing effective interventions that best meet their needs [9].

## Theoretical Framework

**Figure 1:**
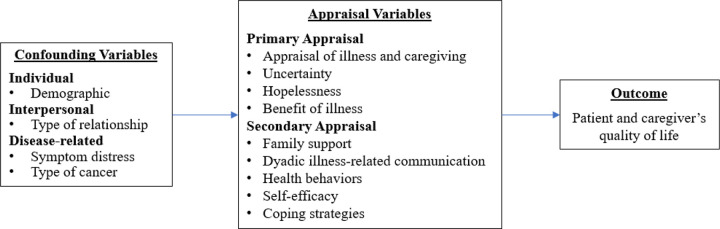
Stress-Coping Theoretical Model adapted from Lazarus and Folkman's (1984) Transactional Model of Stress and Coping (Lewis et al., 2006 & van Lange et al., 2011)

During the past decades, Northouse and colleagues developed a stress-coping model (see Fig. 1) adapted from Lazarus and Folkman’s Transactional Theory of Stress and Coping [10]. The model conceptualizes that an individual’s primary and secondary appraisals influence their QOL.

**Primary appraisals** involve perception and evaluation of the seriousness of the situation, such as advanced cancer and caregiving, and its impact on life and overall well-being. Appraisal of illness and caregiving assesses the potential threats, opportunities, and competing demands, such as the benefit of illness, uncertainties, and feelings of hopelessness [11]. **Secondary appraisals** evaluate an individual’s capabilities and resources to manage the challenges of advanced cancer and related caregiving effectively [11]. These appraisals include health behaviors, self-efficacy, coping strategies, dyadic illness-related communication, and family support.

Northouse’s adapted stress-coping model also conceptualizes that a range of demographic, social, and illness-related variables could influence an individual’s primary and secondary appraisals and, ultimately, the QOL among patients and caregivers managing advanced cancer. Demographic factors (e.g., younger age and lower income and education levels), impaired physical functioning, different types of dyadic relationships (spouse, daughter, son, etc.), and comorbidities and symptoms (e.g., pain and fatigue) have been associated with poorer QOL [12, 13]. Therefore, it is crucial to consider these confounding effects when examining the association between appraisals and QOL in advanced cancer patients and their caregivers.

Our study aimed to examine the associations between primary and secondary appraisals and the QOL while controlling for the confounding effects of demographic, social, and illness-related factors among patients with advanced cancer and their caregivers. We hypothesized that the QOL of patients and their family caregivers is significantly associated with their own primary and secondary appraisal variables. The purpose of this study was to further validate stress-coping theory and inform theory-guided, family-focused, supportive oncologic care for patients with advanced cancer and their family caregivers.

## Methods

### Study Design

This study conducted a secondary analysis of the baseline (Time 1) data collected from a randomized controlled trial (RCT) (R01CA107383, ClinicalTrial.gov: NCT00708968 [PI: Northouse]) that examined the effects of a home-based, dyadic-focused intervention (the FOCUS Program) designed to improve QOL of patients with advanced cancer and their family caregivers [11]. IRB approval was obtained from participating sites and the University of Michigan (IRBMED No. 2004 – 0129).

### Population

In the original RCT, eligible patients needed to meet the following criteria: 1) diagnosed with stage III or IV breast, colorectal, lung, or prostate cancer within the past six months, 2) a life expectancy of at least six months, 3) aged 21 or older, 4) living within 75 miles of participating cancer centers, and 5) having a family caregiver willing to participate. Caregivers were eligible if they were 18 years of age or older, identified as the patient’s primary caregiver, and had not received a cancer diagnosis within the past year or undergoing cancer treatment.

### Setting and Procedures

The setting and procedures of the FOCUS trial were previously published [11]. After clinic staff referred eligible patients and caregivers from four cancer centers who expressed interest in participating, research nurses obtained informed consent and collected the baseline data during a home visit. Data collection occurred at three months and six months post-randomization. We used the baseline data of all study participants to achieve the research aims.

### Measurement

#### Outcome Variable.

The 27-item general Functional Assessment of Cancer Therapy (FACT-G) (version 4) measured **QOL**. This instrument assessed the social, emotional, functional, and physical domains of cancer-related QOL [14]. Caregivers completed a modified version of the FACT-G Caregiver instrument to assess their QOL [15, 16].

#### Primary Appraisal Variables.

Patients completed the 32-item Appraisal of Illness Scale [17] to measure **appraisal of illness.** Caregivers completed the 27-item Appraisal of Caregiving Scale to measure **appraisal of caregiving** [18]. A 9-item brief version of the Mishel Uncertainty in Illness Scale for Adults assessed patient and caregiver **uncertainty** about the disease and their ability to manage it [19]. The 20-item Beck Hopelessness Scale measured **hopelessness**, encompassing three domains: feelings about the future, loss of motivation, and expectations [20]. The 11-item Benefit Finding Scale measured the **benefit of illness**, assessing how attitudes and behaviors changed when one had the illness or cared for someone with it [21].

#### Secondary Appraisal Variables.

The 7-item modified subscale of the Social Support Questionnaire measured **family support** by gauging their perceived social support within the family [22]. The 23-item Lewis Mutuality and Sensitivity Scale [23] evaluated **dyadic illness-related communication** by asking participants to rate their perceived verbal communication, specifically about cancer. A researcher-developed scale analyzed **health behaviors** by requesting patients and caregivers to self-report the frequency of their engagement in exercises and physical activities, balanced nutrition, and adequate sleep [11]. The 17-item Lewis Cancer Self-Efficacy Scale assessed **self-efficacy**, characterized by patient and caregiver confidence in managing cancer [23]. The 38-item Brief Cope evaluated **coping strategies**, which measured how frequently participants used different coping strategies [24]; factor analysis illustrated two subscales: active coping strategies (e.g., use of emotional support) and avoidant coping strategies (e.g., denial).

#### Confounding Variables.

The original study included various individual, interpersonal, and disease-related variables that affect QOL. Patients and caregivers self-reported their age, gender, race, income, and their relationship to each other. The primary RCT obtained the cancer type from the patient’s medical records. The self-administered Risk for Distress Scale (RFD), adapted from the original Omega Clinical Screening Interview, assessed patient and caregiver symptoms separately [25].

### Data Analysis

We conducted a preliminary descriptive analysis to summarize the characteristics of the study participants, including sociodemographic information for patients and caregivers, patients’ medical information, QOL, and their appraisals. We summarized categorical variables using frequencies and percentages and summarized continuous variables using means and standard deviations (SD).

We calculated Pearson’s correlation coefficient between covariates to mitigate unstable coefficient estimates and enhance interpretability by addressing collinearity. In our analysis, none of the correlation coefficients had an absolute value greater than 0.8, so we included all covariates.

To examine the relationships between patients’ and caregivers’ QOL and appraisals while controlling for confounding variables, we conducted multi-level models (MLM) of QOL to account for the nesting of patients and their caregivers within a dyad. We first fitted a full model that included the primary and secondary appraisal variables and the confounders. Interaction terms between role (patient vs. caregiver) and appraisal variables were included to determine if appraisal variables influenced QOL differently for patient and caregiver. The dyad effect was treated as a random effect, and other variables as fixed effects. We fitted the multi-level linear mixed-effects model using the restricted maximum likelihood method (REML) [26]. For the fixed effects, *t*-tests were used to test significance, with Satterthwaite’s method computing denominator degrees of freedom and *t*-value [26].

To simplify the full model and enhance interpretability, we eliminated variables in a backward fashion while respecting the hierarchy of terms. We first tested the interaction terms between role and appraisal variables, avoiding testing corresponding main effects if significant interaction terms were found [26]. Non-significant variables were sequentially eliminated, starting with confounding variables with the smallest effect on QOL, followed by interaction terms and main effects of appraisal variables with the smallest impact. This process continued until a significant difference was observed between the full and selected models by the likelihood-ratio test (*p*-value less than the significance level). The final model was the last selected model that did not differ significantly from the full model in terms of model fitting.

We conducted all statistical analyses with the *R* software. The significance level for all hypothesis testing was set to 0.05.

## Results

### Participants Characteristics

This analysis used baseline data from 362 dyads with complete data for all variables (N = 724 individuals, 362 dyads) (See Table 1). Most caregivers were spouses (73.76%). Most patients (80.4%) and caregivers (80.9%) were non-Hispanic White. The mean age was 60.27 years (SD = 11.60; range 26–95) and 56.45 years (SD = 13.21; range 18–88) for patients and caregivers, respectively. On average, the mean age of patients was more than that of the caregivers (*p* < .05). Regarding income, 53.3% of the patients and 61.3% of the caregivers earned more than $50,000 yearly. Patients had advanced breast (30.94%), colorectal (24.3%), lung (30.7%), and prostate cancer (14.1%). Patients also reported higher symptom distress than caregivers (*p* < .05). Table 2 illustrates the descriptive analysis results of the QOL and appraisal variables for patients and caregivers.

### Associated Factors of QOL Among Patients and Caregivers

The full model included primary and secondary appraisals, role (patient vs. caregivers), interaction terms between role and each appraisal variable, and all confounding variables (see Table 3). We found an association between an improvement in QOL and primary appraisals (less negative appraisals of illness/caregiving [*p* < .0001] and fewer feelings of hopelessness [*p* < .05]) and secondary appraisals (having more family support [*p* < .0001], more frequent engagement in health behaviors [*p* < .001], more use of active coping strategies [*p* < .001], and less reliance on avoidant coping strategies [*p* < .0001]). The only significant interaction terms were role*benefit of illness and role*active coping strategies, indicating that the effects of the benefit of illness (*p* < .05) and active coping strategies on QOL varied between patients and caregivers (*p* < .05).

Additionally, for patients and caregivers, better QOL had a significant correlation with older age (*p* < .0001), being White (*p* < .05), having an income above $50,000 (*p* < .05), and experiencing less symptom distress (*p* < .0001). The role effect demonstrated statistical significance (*p* < .05), indicating that patients had significantly lower QOL than their caregivers when considering appraisals and confounders. We found no significant difference in the association between QOL and type of cancer.

In this full model, the coefficient of determination (*R*^2^) value was 0.7478, indicating that the primary and secondary appraisals can explain about 74.78% of the variance in QOL after controlling for the confounders.

To obtain the final model, guided by the adapted stress-coping model, we conducted a stepwise elimination process for variables with the largest *p*-values in each model, which indicated the smallest effect on QOL (See Table 4). Initially, we focused on confounding variables, starting with the type of cancer. Removing it did not result in a significant difference between the full model and the reduced model (*p* > .05), allowing us to proceed with removing the type of relationship, education, and race, none of which showed significant differences (*p* > .05). However, the removal of gender yielded a significant difference between the full model and the resulting reduced model (*p* < .05), necessitating its retention in the model (fm4). Subsequently, we evaluated interaction terms. We removed six interaction terms until role*active coping strategies cannot be removed (fm11). Lastly, we assessed the main effects of appraisal variables. The removal process was halted when the first variable, dyadic illness-related communication, resulted in a significant difference (*p* < .05), indicating it needed to be retained in the final model (fm11).

As we closely followed the model selection procedures, we observed that removing a variable affected the effects of the remaining variables on the QOL. For example, although race had a significant effect on QOL in the full model (see Table 3), it was removed in Step 4 due to its conditional independence on QOL, given the other remaining variables (see Table 4). This indicated that race appeared significant only when all variables were considered together but became non-significant in fm4. Similarly, role*active coping strategies was significant in the full model but non-significant in fm11. However, it remained in the final model (fm11) because removing it at Step 12 showed a significant difference between the full model and the resulting reduced model. The step-by-step model selection ensured the model's goodness of fit and simplicity.

The final model revealed an association between QOL improvement and primary appraisals (less negative appraisals of illness/caregiving [*p* < .0001] and fewer feelings of uncertainty [*p* < .01] and hopelessness [*p* < .01]) and secondary appraisals (having more family support [*p* < .0001], more frequent engagement in health behaviors [*p* < .0001], higher level of self-efficacy [*p* < .05], more use of active coping strategies [*p* < .01], and less reliance on avoidant coping strategies [*p* < .0001]).

**Figure 2:**
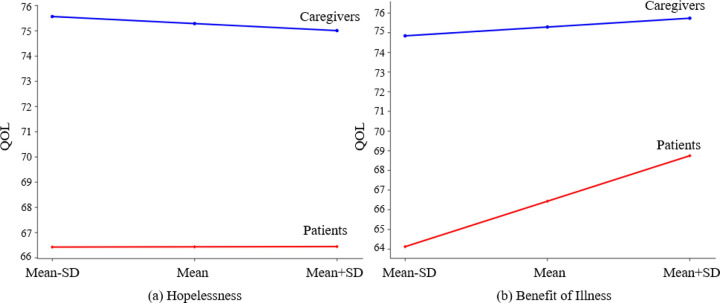
Estimated trajectories of changes in patients’ and caregivers’ QOL. **a.** For patients, the effect of hopelessness on QOL is −0.41+0.43 = 0.02, indicating that as the hopelessness in patients increased, their QOL increased; while for caregivers, the effect of hopelessness on QOL is −0.41, indicating that as the hopelessness in caregivers increased, the QOL decreased. Note: a higher score on the hopelessness scale indicates more hopelessness is perceived. **b.** For patients, the effect of the benefit of illness on QOL is 0.66+2.75 = 3.41, indicating that as the benefit of illness in patients increases by one unit, their QOL increases by 3.41 units. For caregivers, the effect of the benefit of illness on QOL is 0.66, indicating that as the benefit of illness in caregivers increases by one unit, their QOL increases by 0.66 unit. Note: a higher score on the benefit of illness scale indicates more benefit of illness is perceived.

The only significant interaction terms were role*hopelessness and role*benefit of illness (see Fig. 2), indicating that the associations between QOL and hopelessness and the benefit of illness varied between patients and caregivers. A one-unit increase in hopelessness among patients was associated with an increase in QOL, while as the hopelessness in caregivers increased, the QOL decreased. Compared to caregivers, a one-unit increase in the benefit of illness was associated with a substantial QOL improvement among patients (both *p*s < .05). The effects of role*family support and role*active coping strategies were marginally significant (*p*s = 0.08 and 0.09, respectively).

Among all confounding variables, better QOL was significantly associated with older age (*p* < .0001), having an income above $50,000 (*p* < .05), and experiencing less symptom distress (*p* < .0001). Furthermore, the role effect on QOL became non-significant, suggesting the effect of role was masked by the interaction effects between appraisals and roles. The appraisals (how they evaluate their circumstances) interacted with their roles in a way that hid the direct effect of the role on QOL. In simpler terms, how patients and caregivers appraised their situation could overshadow the direct influence that their specific role might have on their QOL.

In the final model, the coefficient of determination (*R*^2^) value was 0.7414, indicating approximately 74.14% of the variance in QOL was explained by the primary appraisals (appraisals of illness/caregiving, uncertainty, hopelessness, benefit of illness, role*hopelessness, and role*benefit of illness) and secondary appraisals (family support, dyadic illness-related communication, health behaviors, self-efficacy, active and avoidance coping strategies, role* family support, and role*active coping strategies) after controlling for the effects of the confounders (age, gender, income, and symptom distress) and role.

## Discussion

This study is among the few theory-guided studies to comprehensively examine the associations between primary and secondary appraisals and QOL among patients with advanced cancer and their family caregivers using a large sample. Supporting the adapted stress-coping model by Northouse et al, this study found that better QOL for patients with advanced cancer and their family caregivers was significantly associated with their primary appraisals (appraisals of illness/caregiving, uncertainty, hopelessness) and secondary appraisals (family support, health behaviors, self-efficacy, and active and avoidant coping strategies) while controlling for confounding variables (age, income, and symptom distress). Additionally, the associations between QOL and factors like hopelessness and the benefit of illness varied between patients and caregivers. Together with several non-significant variables, these appraisal factors and confounding variables explained more than 74% of the variance in QOL.

Our study emphasizes the explanatory power of Northouse's adapted stress-coping model in capturing key factors affecting QOL for patients and caregivers managing advanced cancer. It highlights the model's usefulness in understanding and addressing these challenges. This study provides compelling evidence of the role of perception and coping strategies in determining QOL during advanced cancer survivorship. By including both patients and caregivers, this research broadens our understanding of how stress-coping and psychosocial support, alongside medical treatment, enhance QOL for those managing advanced cancer.

### Associations Between Primary Appraisal Variables and QOL

Our study found that primary appraisals were associated with QOL. Primary appraisals evaluate whether a situation that threatens well-being is manageable, or is benign or advantageous [10]. Corroborating previous research [27], we found that less negative appraisals of illness/caregiving were associated with higher QOL. Patients may view physical and psychological symptoms as signs of declining health or increased dependency, negatively impacting their QOL. Similarly, caregivers may see daily responsibilities as burdensome, further diminishing their QOL [28]. These perspectives underscore the importance of supportive care that addresses their perceptions and experiences during advanced cancer.

We found negative associations between QOL and uncertainty and hopelessness, significant sources of psychological distress for cancer patients and their families. Other studies have reported similar findings but without considering caregivers/families as subjects [29]. A recent study demonstrated that hope and uncertainty accounted for 22% of the variance in anxiety and 34% of depressive symptoms among patients with advanced lung cancer, highlighting the clinical relevance of addressing these factors in psychological intervention [30]. Our study expands on prior research by examining the roles of uncertainty and hopelessness on the overall well-being of both patients and caregivers.

We identified differences in the associations between QOL and hopelessness for patients and caregivers. Specifically, patients had worse QOL than caregivers, but their QOL was less affected by hopelessness, possibly indicating psychological adaptation among patients. In contrast, caregivers may be more emotionally vulnerable due to the patient’s disease progression and anticipated loss of their loved one, making their QOL more sensitive to hopelessness [31].

Additionally, we discovered that the associations between QOL and the benefit of illness varied between patients and caregivers. While the increased perceived benefit of illness improved QOL for both, the positive impact was more significant for patients, indicating psychological resilience, which positively affects patients’ QOL [32]. Caregivers’ perceived benefits had less impact on their QOL due to other responsibilities or stressors. These findings highlight the importance of considering the unique experiences and perceptions of both patients and caregivers in advanced cancer settings.

### Associations Between Secondary Appraisal Variables and QOL

Our study demonstrated that almost all secondary appraisals were associated with QOL. Secondary appraisals evaluate an individual’s resources and capability to cope [10]. Family support was significantly associated with QOL for patients and caregivers. In a previous study, patients with advanced colorectal cancer and their caregivers viewed strengthened relationships with family and friends as a significant benefit of the illness, improving QOL through practical, emotional, and spiritual support [33].

Health behaviors are active coping strategies for advanced cancer patients and their family caregivers. Our study found that better QOL was associated with more frequent engagement in health behaviors. However, patients with advanced cancer might be less inclined to adopt lifestyle changes due to physical limitations. In contrast, caregivers facing fewer physical constraints may be able to engage in these behaviors [34]. Despite multiple demands, such as employment, family responsibilities, and caregiving, caregivers may try to cope with the distress by actively engaging in health behaviors. This discrepancy emphasizes the complexity of applying a one-size-fits-all approach to health behaviors in advanced cancer.

Self-efficacy was also linked to QOL, indicating that believing in one's abilities to accomplish tasks, achieve goals, or handle challenges successfully was beneficial for both patients and caregivers [35]. Our findings align with recent reviews that reported significant associations between lower self-efficacy, greater distress, and poorer QOL [36]. Our study adds evidence of significant associations between secondary appraisals and QOL in caregivers to the literature.

Active coping was positively associated with QOL for patients and caregivers, whereas avoidant coping was negatively associated. Our findings are consistent with recent reviews indicating that avoidant coping and greater distress were associated with poorer QOL [36]. Patients with advanced cancer are more likely to adopt active coping strategies, such as seeking support from family and friends, possibly due to the increased availability of caregivers in this setting compared to curative care, ultimately improving the patients' QOL [37].

### Associations Between Confounding Variables and QOL

In addition, we found a significant association between several demographic and illness-related factors and better QOL of patients and their caregivers: older age, better income, and less symptom distress. Previous studies have shown that younger cancer patients experience more significant financial difficulties and challenges with social and role functioning [38]. Patients with lower income have poorer QOL and survival [12]. The presence and severity of physical symptoms, such as pain, fatigue, nausea, and breathlessness, are likely to negatively impact patient well-being [13].

### Study Limitations

This study has several limitations that warrant further research. First, it used a cross-sectional design to maximize the sample size for a robust analysis of many theoretical constructs as conceptualized in Northouse’s adapted stress-coping model while teasing out the intervention effects on QOL. Future research could explore more complex dyadic patterns by utilizing longitudinal dyadic data. Furthermore, the lack of sociodemographic diversity in the dataset, which was predominantly non-Hispanic Whites, necessitates broader exploration within more diverse populations, particularly minority Hispanics. They may face distinct linguistic and cultural challenges during advanced cancer, which could impact their perceptions and evaluations of the stressor and their coping abilities. Moreover, this study did not examine the potential interdependent process of stress coping. Our current research did not explore how patients’ stress-coping strategies influence caregivers’ QOL or vice versa. Finally, this study focused on the QOL total score, encompassing physical, social, emotional, and functional subdomains. Future dyadic research can investigate various forms of interdependence within the domains of QOL [39] and directional effects with latent constructs and measurement models.

### Clinical and Research Implications

Our study examined the adapted stress-coping framework and provided crucial insights for shaping future research design and intervention development to support individuals managing advanced cancer.

1. Interventions should target both patients with advanced cancer and their caregivers [40], recognizing the likely decline in their QOL and the shared psychosocial challenges they face managing advanced cancer.
2. Future interventions should prioritize and include vital components such as addressing negative appraisals of illness and caregiving, reducing uncertainty and hopelessness, bolstering family support, promoting health behaviors and self-efficacy, and refining active and avoidant coping strategies to optimize QOL for patients and caregivers.
3. Future interventions must be customized to accommodate patients' and caregivers' unique needs and experiences, recognizing that factors such as hopelessness and perceived benefit of illness may impact them differently. This tailored approach ensures interventions are sensitive to each group's specific needs within the advanced cancer framework.

## Conclusion

To our knowledge, our study represents the first application of a theory-driven approach to conducting a comprehensive, robust analysis of the associations between QOL and a full range of primary and secondary appraisal factors with a notably large sample of patients with advanced cancer and their family caregivers using multi-level analyses. Our findings indicate how patients and caregivers perceive and respond to the challenges of advanced cancer and how they influence their QOL. It is crucial to tailor these interventions to meet the specific needs of patients with advanced cancer and caregivers.

## Figures and Tables

**Table 1:** Descriptive statistics for participants, their quality of life, and appr (N = 724 subjects, 362 dyads)

Characteristics	Patient (N = 362)		Caregiver (N = 362)		
	Mean	SD	Mean	SD	*p*-value
**Age (Year)**	60.27	11.60	56.45	13.21	**< .05** *
**Education (Year)**	14.64	2.82	14.73	2.86	.49
**Mean symptom distress**	11.22	5.14	6.82	6.90	**< .05** *
	**N**	**%**	**N**	**%**	**p-value**
**Gender**					.55
Male	146	40.3	155	42.8	--
Female	216	59.7	207	57.2	--
**Race**					.93
White	291	80.4	293	80.9	--
Non-White	71	19.6	69	19.1	--
**Ethnicity**					1
Hispanic	6	1.7	7	1.9	--
Non-Hispanic	356	98.3	355	98.1	--
**Income**					< .05*
<= $ 50,000	169	46.7	140	38.7	--
> $ 50,000	193	53.3	222	61.3	--
**Type of relationship**					--
Spouse	267	73.8	--	--	--
Non-Spouse	95	26.2	--	--	--
**Type of cancer**					--
Breast Cancer	112	30.9	--	--	--
Colorectal Cancer	88	24.3	--	--	--
Lung Cancer	111	30.7	--	--	--
Prostate Cancer	51	14.1	--	--	--

Note:

1. Percentages have been rounded and may not total 100.

2. The *p*-values of categorical variables were calculated based on the chi-squared test. The *p*-values of continuous variables were obtained from the paired *t*-test.

* indicates the *p*-value smaller than 0.05, which is considered significant.

**Table 2:** Descriptive statistics of QOL and appraisal variables for patients and caregivers

Construct Variables	Cronbach’s Alpha		Mean (SD)		*p*-value
	Patients	Caregivers	Patients	Caregivers	
Quality of life ^†^	0.72 ^§^	0.74 ^§^	76.04 (16.69)	76.50 (15.17)	.62
Appraisal of illness/caregiving ^‡^	0.94	0.87	3.25 (0.72)	2.87 (0.53)	< .05*
Uncertainty ^‡^	0.75	0.71	20.27 (4.81)	20.17 (4.51)	.73
Hopelessness ^‡^	0.86	0.83	4.77 (4.09)	4.54 (3.68)	.35
Benefit of illness ^†^	0.90	0.91	3.10 (0.63)	2.84 (0.69)	< .05*
Family support ^†^	0.84	0.85	4.26 (0.68)	3.97 (0.75)	< .05*
Dyadic illness-related communication ^†^	0.93	0.93	82.74 (15.80)	81.44 (15.71)	.17
Health behaviors ^†^	0.57	0.68	27.89 (6.92)	26.21 (7.87)	< .05*
Self-efficacy ^†^	0.97	0.97	132.15 (29.43)	132.05 (27.90)	1
Coping strategies					
Active coping strategies ^†^	0.87	0.87	2.85 (0.55)	2.66 (0.54)	< .05*
Avoidant coping strategies ^‡^	0.79	0.74	1.56 (0.52)	1.52 (0.46)	.26

Note:

†: Higher scores indicated more positive results, i.e., better quality of life, more benefit of illness, more family support, better dyadic illness-related communication, more frequent engagement in health behaviors, and more active coping strategies adopted (e.g., getting advice or help);

‡: Higher scores indicated more negative results, i.e., more negative appraisal of illness/caregiving as a threat, more feelings of uncertainty and hopelessness, and more avoidant coping strategies adopted (e.g., alcohol or drug use).

§: The Cronbach’s alpha was calculated based on the 4 subscales of the Functional Assessment of Cancer Therapy (FACT-G) among patients and caregivers.

* : Significant findings.

**Table 3:** Full multilevel model and final model of QOL with interaction terms of role and appraisal variables

	Full Model			Final Modal		
Effect	Estimate	SE	*p*-value	Estimate	SE	*p*-value
Intercept	85.74	7.42	**< .0001**	75.29	5.40	**< .0001**
Role (referent: caregiver)	−20.20	9.24	**0.0292**	−8.85	5.64	0.1172
**Primary Appraisals**						
Appraisal of illness/caregiving ^†^	−7.63	1.32	**< .0001**	−6.48	0.78	**< .0001**
Uncertainty ^†^	−0.22	0.14	0.1170	−0.28	0.10	**0.0057**
Hopelessness ^†^	−0.42	0.16	**0.0115**	−0.41	0.15	**0.0047**
Benefit of illness ^‡^	0.79	0.81	0.3283	0.66	0.77	0.3912
**Secondary Appraisals**						
Family support ^‡^	3.68	0.89	**< .0001**	3.48	0.76	**<. 0001**
Dyadic illness-related communication ^‡^	−0.07	0.04	0.0918	−0.05	0.03	0.1264
Health behaviors ^‡^	0.24	0.06	**0.0002**	0.32	0.05	**< .0001**
Self-efficacy ^‡^	0.02	0.02	0.3206	0.04	0.02	0.0116
Coping strategies						
Active coping strategies ^‡^	3.62	1.04	**0.0005**	2.87	0.99	**0.0037**
Avoidant coping strategies ^†^	−9.67	1.28	**< .0001**	−7.78	0.84	**< .0001**
**Confounding Variables**						
Age	0.16	0.03	**< .0001**	0.16	0.03	**< .0001**
Gender (referent: female)	−0.96	0.69	0.1646	−1.13	0.68	0.0942
Race (referent: non-White)	1.84	0.92	**0.0477**			
Education	−0.18	0.14	0.1877			
Income (referent: <= $ 50,000)	1.79	0.82	**0.0289**	1.56	0.70	**0.0267**
Type of relationship (referent: non-spouse)	−1.22	0.88	0.1657			
Symptom distress	−0.51	0.06	**< .0001**	−0.54	0.06	**< .0001**
Type of cancer (referent: breast cancer)						
Colorectal cancer	−1.08	0.92	0.2424			
Lung cancer	−0.88	0.89	0.3261			
Prostate cancer	1.36	1.15	0.2357			
**Interaction Terms**						
Role * Appraisal of illness/caregiving	1.43	1.59	0.3694			
Role * Uncertainty	−0.11	0.20	0.5831			
Role * Hopelessness	0.40	0.23	0.0772	0.43	0.18	**0.0173**
Role * Benefit of illness	2.34	1.17	**0.0456**	2.75	1.12	**0.0145**
Role * Family support	0.86	1.28	0.5014	1.61	0.93	0.0844
Role * Dyadic illness- related communication	0.05	0.06	0.4179			
Role * Health behaviors	0.15	0.09	0.1020			
Role * Self-efficacy	0.03	0.03	0.3415			
Role * Coping strategies						
Role * Active coping strategies	−3.37	1.43	**0.0188**	−2.28	1.33	0.0878
Role * Avoidant coping strategies	2.96	1.66	0.0749			

Note:

1. The coefficient of determination (*R*^2^) values of the full model and final model are 0.7478 and 0.7414, respectively.

2. The *p*-value of the likelihood-ratio test comparing the full model and final model is 0.0748.

3. †: Higher scores indicated more negative results, i.e., more negative appraisal of illness/caregiving as a threat, more feelings of uncertainty and hopelessness, and more avoidant coping strategies adopted (e.g., alcohol or drug use).

4. ‡: Higher scores indicated more positive results, i.e., more benefit of illness, more family support, better dyadic illness-related communication, more frequent engagement in health behaviors, and more active coping strategies adopted (e.g., getting advice or help).

**Table 4:** Step-by-step procedures of model selection

Variables	ANOVA Chi-Square	Degree of Freedom	*P*-Value†	R^2^	Results	Model Selected
**Confounding Variables**						
1 Type of cancer	5.84	3	0.1198	0.7472	Type of cancer is removed.	Fm1
2 Type of relationship	7.37	4	0.1178	0.7472	Type of relationship is removed.	Fm2
3 Education	9.30	5	0.0978	0.7463	Education is removed.	Fm3
4 Race	12.31	6	0.0555	0.7448	Race is removed.	Fm4
5 Gender	15.67	7	**0.0283** *	0.7457	Gender needs to be kept.	Fm4
After removing “gender,” a significant difference is observed between the full model and model fm4 (ρ < .05); therefore, “gender” and other remaining confounding variables need to be kept in the final model. Confounding variables of type of cancer, type of relationship, education, and race are removed from the model. The selection of confounding variables stops.						
**Interaction Terms**						
6 Role*uncertainty	12.55	7	0.0838	0.7449	Role*uncertainty is removed.	Fm6
7 Role* dyadic illness-related communication	13.18	8	0.1058	0.7444	Role*dyadic illness-related communication is removed.	Fm7
8 Role*appraisal of illness/caregiving	13.94	9	0.1245	0.7438	Role*appraisal of illness/caregiving is removed.	Fm8
9 Role*self-efficacy	14.76	10	0.1409	0.7434	Role*self-efficacy is removed.	Fm9
**Confounding Variables**						
10 Role*avoidant coping strategies	16.98	11	0.1085	0.7414	Role*avoidant coping strategies is removed.	Fm10
11 Role*health behaviors	19.61	12	0.0748	0.7414‡	Role*health behaviors is removed.	Fm11
12 Role*active coping strategies	22.61	13	**0.0467** *	0.7394	Role*active coping strategies needs to be kept.	Fm11
After removing “role*active coping strategies”, a significant difference is observed between the full model and model fm11 (ρ < .05); therefore, “role*active coping strategies” and other remaining interaction terms need to be kept in the final model. Interaction terms of role*uncertainty, role*dyadic illness-related communication, role*appraisal of illness/caregiving, role*self-efficacy, role*avoidant coping strategies, and role*health behaviors are removed from the model. The selection of interaction terms stops.						
**Main Effects of the Appraisal Variables**						
13 Dyadic illness-related communication	24.84	14	0.0362	0.7379	Dyadic illness-related communication needs to be kept.	Fm11
After removing “dyadic illness-related communication,” a significant difference is observed between the full model and model fm11 (ρ < .05); therefore, “dyadic illness-related communication” and other main effects of the appraisal variables need to be kept in the final model. No main effect of the appraisal variable is removed from the model. The model selection stops. Fm11 is selected as the final model.						

Note:

1. †: *ρ*-value is the result of the likelihood-ratio test comparing the full and selected models.

2. ‡: R^2^ is 0.7414 for the final model, which is fm11, indicating the variables in the final model explained 74.14% of the variance in the QOL of patients with advanced cancer and their caregivers.

3. *: The *p*-values of the likelihood-ratio test comparing the full model and selected models are < 0.05, and the model selection for that group of variables is complete.
